# Editorial: Enhancing food security through sustainable diets and postharvest innovation in low- and middle-income countries

**DOI:** 10.3389/fnut.2026.1836309

**Published:** 2026-04-20

**Authors:** Chala G. Kuyu, Mohamed H. H. Roby, Haile Tesfaye Duguma, Tamiru Yazew

**Affiliations:** 1 Department of Postharvest Management, Jimma University College of Agriculture and Veterinary Medicine, Jimma, Ethiopia; 2 Food Science and Technology Department, Faculty of Agriculture, Fayoum University, Fayoum, Egypt; 3 School of Packaging, Michigan State University, East Lansing, MI, United States; 4 Department of Public Health, College of Health Sciences, Salale University, Fitche, Ethiopia

**Keywords:** food security, loss reduction, low- and middle-income countries, nutrient-dense food, sustainability

The global food system faces intensifying pressures from climate change, resource scarcity, and persistent malnutrition, demanding novels, integrated strategies to ensure both sustainability and nutritional security. This Research Topic synthesizes diverse approaches to these challenges, examining how maximizing the utility of resilient crops and minimizing post-harvest loss (PHL) can substantially enhance food availability and public health outcomes particularly in low- and middle-income countries. The research collectively frames the urgent need for interventions across the value chain, from optimal harvesting practices for staples to developing nutrient-dense alternatives and addressing behavioral waste at the household level.

The current Research Topic is aimed to contribute empirical evidence toward developing resilient, diversified, and nutrition-sensitive food systems. This includes: (1) evaluating climate-resilient crops as alternatives to vulnerable staples like wheat; (2) quantifying the magnitude and nutritional consequences of post-harvest loss in perishable foods; and (3) optimizing mechanical harvesting processes to reduce waste and environmental impact in major grain production. By integrating agricultural efficiency, nutritional science, and public health interventions, this Research Topic provides a multi-pronged perspective on fortifying food security for vulnerable populations.

After 8 months' call for contributions, five papers of pertinence to the Research Topic were accepted as publications on *Frontiers in Nutrition*. All the reported scientific discoveries addressed empirical studies, mechanistic research, and trials that examine how interventions across the value chain starting from harvest to consumption can improve food availability, retention of nutrients, and equitable nutrition outcomes. An evidence emerging from this topic shows that affordable innovations can mitigate loss and create economic incentives that strengthen food security.

In low- and middle-income countries limited availability and access to nutrient-dense foods contribute to malnutrition, micronutrient deficiencies, and poor health outcomes, particularly among vulnerable populations such as children and women. A new evidence from Fatima et al. show that profiling and testing new sorghum-soy composite snacks, nutrient-dense, gluten-free products have a potential to diversify diets while delivering higher protein and lower glycemic responses. This study focused on developing high-protein, low-glycemic, and gluten-free chips using sorghum and soy flours, highlighting the shift needed toward diverse, nutrient-dense, and climate-resilient crops. Wheat production is increasingly threatened by climate change, underscoring the value of alternatives like sorghum and soybeans, which are rich in fiber, quality proteins, and adaptable to marginal soils. The research investigated four formulations of sorghum-soy chips processed through baking and frying. The formulation with a 55:45 soybean: sorghum ratio (T1) received the highest sensory scores, demonstrating strong consumer preference. Proximate analysis confirmed that increasing soybean incorporation led to significant increases in protein content, especially in T3 (65:35), and provided appreciable levels of essential minerals like calcium, iron, and magnesium. The baked chips exhibited a lower glycemic index and reduced fat content, making them appropriate for diabetic or health-conscious consumers, while fried variants offered superior texture and shelf stability. The principal component analysis (PCA) visually demonstrated the compromise between nutritional quality (favored by baking) and sensory acceptability (favored by frying).

Sub-Saharan Africa loses reach up to 50% of fruits and vegetables produced annually, threatening nutrition security by reducing the availability of micronutrient-rich foods. Study protocol conducted by Twum-Dei et al. discussed the design for a community-based randomized trial in Ghana to explicitly test relationships between post-harvest loss of fruits and vegetables. It details protocols to measurable nutritional outcomes in women and young children, an approach that bridges food systems loss and human nutrition. This manuscript details the protocol for a community-based randomized controlled trial in the Ho West District of Ghana, designed to systematically investigate the link between post-harvest losses (PHL) of fruits and vegetables and nutritional deficiencies in vulnerable groups. The protocol aims to estimate the extent and drivers of PHL across the value chain and evaluate the effect of a 6-month structured nutrition education and counseling intervention on reducing waste and improving nutritional outcomes, such as dietary diversity and anemia status. The intervention is grounded in the Health Belief Model and Social Cognitive Theory to promote sustained behavior change in storage and handling practices.

Another study by Sun et al., addressed PHL in major staple crops by focusing on mechanical harvesting of rice in China, where rice output accounts for over 40% of the national total in Heilongjiang Province. The study aimed to enhance mechanical efficiency and reduce rice yield loss during harvesting, which concurrently helps lower associated carbon emissions (15–20% of the entire planting process). Using single-factor ANOVA and correlation analysis, the study determined optimal harvesting conditions: grain moisture content of 20–22%, a lower harvester traveling speed (5–6 km/h), and an appropriate stubble height (14–18 cm). Findings showed that adhering to these optimal parameters can reduce rice grain shedding by approximately 1–1.5% and prevent increases in fuel consumption and CO_2_ emissions associated with high-speed operations. The study also highlighted that storage loss, though lowest with three daily turnovers, is significantly affected by the moisture content of the stored rice.

In summary the above research articles mentioned that addressing food insecurity in LMICs requires more than increasing production; it demands a food systems approach that reduces losses of nutrient-rich foods along value chains, develops affordable and nutritious products from local crops, and links these interventions to measurable nutrition and public health outcomes, particularly for women and children. By integrating post-harvest innovation with diet quality strategies, these efforts support sustainable, equitable, and resilient food systems in line with global frameworks like the Sustainable Development Goals. Future efforts, as suggested by these findings, should focus on market feasibility and policy alignment to ensure scalable production of nutrient-dense alternatives, extended observation periods for PHL intervention studies, and farmer education regarding the technical nuances of loss reduction in mechanical harvesting. By connecting agricultural practices (PHL mitigation) with public health outcomes (improved nutritional status), this Research Topic offers a robust framework for strengthening resilient and sustainable food systems globally.

